# Targeted sequencing reveals the mutational landscape responsible for sorafenib therapy in advanced hepatocellular carcinoma

**DOI:** 10.7150/thno.41616

**Published:** 2020-04-06

**Authors:** Jing Tang, Cheng-Jun Sui, Dong-Fang Wang, Xin-Yuan Lu, Gui-Juan Luo, Qing Zhao, Qiu-Yu Lian, Seogsong Jeong, Xi-Meng Lin, Yan-Jing Zhu, Bo Zheng, Rui Wu, Qing Wang, Xiao-Long Liu, Jing-Feng Liu, Qiang Xia, Gang Wu, Jin Gu, Hong-Yang Wang, Lei Chen

**Affiliations:** 1 Cancer Center, Union Hospital, Tongji Medical College, Huazhong University of Science and Technology, Wuhan 430022, China.; 2 The International Cooperation Laboratory on Signal Transduction, Eastern Hepatobiliary Surgery Hospital, Second Military Medical University, Shanghai 200438, China.; 3 Department of special treatment and liver transplantation, Eastern Hepatobiliary Surgery Hospital, Second Military Medical University, Changhai Road 225, Shanghai 200438, China; 4 MOE Key Laboratory for Bioinformatics, BNRIST Bioinformatics Division, Department of Automation, Tsinghua University, Beijing 100084, China; 5 Department of Pathology, Eastern Hepatobiliary Surgery Hospital, Second Military Medical University, Changhai Road 225, Shanghai, Shanghai 200438, China; 6 Department of Clinical Pharmacology, Xiangya Hospital, Central South University, Changsha, Hunan, China. 2Institute of Clinical Pharmacology, Central South University, Changsha, Hunan 410013, China.; 7 Department of Liver Surgery, Renji Hospital, School of Medicine, Shanghai Jiao Tong University, Shanghai, 200127, China.; 8 Department of Biliary Surgery I, Eastern Hepatobiliary Surgery Hospital, Second Military Medical University, Changhai Road 225, Shanghai 200438, China; 9 The United Innovation of Mengchao Hepatobiliary Technology Key Laboratory of Fujian Province, Mengchao Hepatobiliary Hospital of Fujian Medical University, Fuzhou 350025, China; 10 Fudan University Shanghai Cancer Center; Department of Oncology, Shanghai Medical College, Fudan University, Shanghai 200032, China; 11 Ministry of Education (MOE) Key Laboratory on signaling Regulation and Targeting Therapy of Liver Cancer, Shanghai 200438, China; 12 Shanghai Key Laboratory of Hepato-biliary Tumor Biology, Shanghai 200438, China

**Keywords:** Mutational Landscape, Sorafenib Resistance, *TP53*, * OCT4*, Hepatocellular Carcinoma.

## Abstract

**Rationale**: The existence of primary and acquired drug resistance is the main obstacle for the effect of multi-kinase inhibitor sorafenib and regorafenib in advanced hepatocellular carcinoma (HCC). However, plenty of patients did not significantly benefit from sorafenib treatment and little is known about the mechanism of drug resistance.

**Methods**: Laser capture microdissection was used to acquire matched normal liver and tumor tissues on formalin-fixed paraffin-embedded specimens collected before sorafenib therapy from the first surgery of 119 HCC patients. Ultra-deep sequencing (~1000×) targeting whole exons of 440 genes in microdissected specimens and siRNA screen in 7 cell lines were performed to find mutations associated with differential responses to sorafenib. Patient-derived xenograft models were employed to determine the role of *TP53* in response to sorafenib. Lentiviruses harboring wild-type and c.G52C-mutant *OCT4* were applied to explore the function of* OCT4* in resistance to sorafenib. ChIP-PCR assay for analysis of OCT4 transcriptional activity was performed to explore the affinity with the* KITLG* promoter. Statistical analyses were used to associate levels of p53 and OCT4 with tumor features and patient outcomes.

**Results**: Total 1,050 somatic mutations and 26 significant driver genes were identified. SiRNA screening in 7 HCC cell lines was further performed to identify mutations associated with differential responses to sorafenib. A recurrent nonsynonymous mutation c.G52C in *OCT4* (*OCT4*^mut^) was strongly associated with good response to sorafenib, whereas the stop-gain mutation in *TP53* showed the opposite outcome both in vitro and in vivo. *OCT4*^wt^-induced stem cell factor (encoded by *KITLG* gene, SCF) expression and cross-activation of c-KIT/FLT3-BRAF signals were identified indispensably for sorafenib resistance, which could be reversed by the combination of c-KIT tyrosine kinase inhibitors or neutralizing antibody against SCF. Mechanistically, an OCT4 binding site in upstream of *KITLG* promoter was identified with a higher affinity to wildtype of OCT4 rather than G52C-mutant form, which is indispensable for OCT4-induced expression of *KITLG* and sorafenib resistance.

**Conclusion**: Our study reported a novel somatic mutation in *OCT4* (c.G52C) responsible for the sorafenib effect, and also shed new light on the treatment of HCC through the combination of specific tyrosine kinase inhibitors according to individual genetic patterns.

## Introduction

Hepatocellular carcinoma (HCC) is one of the most common malignancies and lethal neoplasm worldwide with rapidly increasing incidence 1. Despite recent advances in surgical techniques and the discovery of various therapeutic strategies, the prognosis of HCC remains dismal 2. Curative modalities such as radical resection and transplantation, cannot be used in the majority of patients with HCC due to the rapid progression of the tumor and its advanced stage at the time of diagnosis 3. For those patients, besides several immunotherapeutic strategies, the multikinase inhibitors, such as sorafenib and regorafenib are the major drugs for the treatment of advanced HCC (aHCC) clinically 4.

Sorafenib was firstly approved for the treatment of HCC by the European Medicines Agency and U.S. Food and Drug Administration in the last decade, which was supported by the results of a large-scale, multi-institutional, double-blind, placebo-controlled phase III clinical trial that included 602 patients with aHCC 5. The Sorafenib Hepatocarcinoma Assessment Randomized Protocol (SHARP) study demonstrated a remarkably significant increase in OS (median survival months, 10.7 vs. 7.9) and time to radiologic progression (median progression-free months, 5.5 vs. 2.8) when the patients received sorafenib treatment. However, low overall response rate and acquired drug resistance are the major factor hampering the usage of sorafenib clinically and has raised global concern for understanding the underlying mechanism 6.

Previous studies have documented that the outcomes of sorafenib treatments are partly dependent on the BCLC stage, Child-Pugh score, performance status of the patient 7, and the basal levels of pERK, JNK or VEGFA in HCC tissues. In addition, the crosstalk involving the PI3K/AKT and JAK-STAT pathways, the activation of hypoxia-inducible pathways, epithelial-mesenchymal transition, epigenetic regulation, and tumor environment were also involved in sorafenib resistance. Although many genetic features related to drug response have been intensively investigated in different types of cancer by applying high-throughput sequencing techniques 8, 9, the recurrent genetic variations associated with sorafenib resistance in aHCC are still uncovered. Here, we selected 440 genes including oncogenes, tumor suppressors and immuno-genes, and analyzed potential genetic variations related to sorafenib resistance by applying ultra-deep whole exons sequencing method with FFPE specimens from 119 HCC cases.

## Methods

### Patients and samples

HCC patients diagnosed for the first time at Eastern Hepatobiliary Surgery Hospital (EHBH) and underwent hepatectomy during 2006-2013. A total of 119 post-resection patients with recurrent HCC who received systemic therapy containing sorafenib were enrolled. FFPE specimens derived from hepatectomy were serially cut onto glass slides or polyethylene naphthalate membrane slides (Leica). Hematoxylin & Eosin staining was then performed to determine the distribution of tumor and normal tissues by three pathologists and laser capture of histologically matched tumor and normal tissue was performed using an LS-AMD microscope (Leica) according to the manufacture's protocol. Demographic and clinical characteristics of these patients are shown in - Table S1 and S2. All patients provided written informed consent for treatment and molecular analysis, with ethical approval provided by the EHBH Ethics Committee. The therapeutic effect of sorafenib was assessed according to the mRECIST criteria 10.

### DNA targeted sequencing

We used 119 pairs of tumors and normal FFPE specimens, seven PDX tissues and seven HCC cell lines for targeted sequencing. Isolated genomic DNA concentration was verified using the Qubit dsDNA HS Assay Kit (Life Technologies, Carlsbad, California, USA). Samples were prepared as described in the Ion AmpliSeq Library Preparation User Guide, Publication #MAN0006735 Rev. 5.0 (Life Technologies) using the Ion AmpliSeq Library Kit 2.0 with Ion AmpliSeq Comprehensive Cancer Panel v2.0 (including 409 genes, Life Technologies) and our custom-designed Panel (31 HCC related genes). Ion Xpress Barcode Adapters 1-64 Kit (Life Technologies) was used during the adapter ligation step of the library preparation to uniquely barcode each sample in one single run. Following option 2 of the user guide, libraries were quantified by qPCR. Libraries were diluted to 100 pM based on molarity values from qPCR assay before pooling. An equimolar mix of barcoded libraries was prepared and then diluted to 10 pM. The 10 pM library pool was used in the preparation of template-positive Ion Sphere Particles (ISPs) containing clonally amplified DNA using the Ion PI™ Template OT2 200 Kit v3 on the Ion OneTouch 2 System (Life Technologies). Template-positive ISPs were enriched using the Ion OneTouch ES all as described in the Ion PI™ Template OT2 200 Kit v3 User Guide, Publication #MAN0009133 Rev. B.0 (Life Technologies). Enriched ISPs were loaded onto an Ion PI™ Chip v2 and sequenced with the Ion PI™ Sequencing 200 Kit v3 on an Ion Proton System as described in the Ion PI™ Sequencing 200 Kit v3 User Guide, Publication #MAN0009136 Rev. B.0 (Life Technologies).

### Variant calling, filtering, and annotation

The raw sequence data were processed by Torrent Suite Software (Life Technologies). Candidate somatic mutations were identified by comparing the called variants from the matched tumors and adjacent normal tissues with a minimal mutation rate of 5% (this cutoff was chosen by considering the high sequencing depth). Then, stringent filtering steps were used to reduce the false positives: 1) only point mutations were kept (due to the high error rate of polymers by Ion Proton); 2) the sites, which are heterozygous in adjacent normal tissues, were removed (exclude the effects of CNVs or mixture populations); and 3) all the sites overlapped with known SNPs (dbSNP and 1000g) were excluded. The functional impacts of the identified mutations were annotated by ANNOVAR^11^. The driver genes were analyzed by MutSigCV 12.The sequence data can be downloaded via Genome Sequence Archive (GSA) Accession CRA001003 (Temporary link for review: [http://bigd.big.ac.cn/gsa/s/dI7yD1ja]).

### Statistical analysis

Demographic and clinical characteristics were presented as median (interquartile range [IQR]). Cox proportional hazards model was applied to evaluate the hazard ratio (HR) with 95% confidence interval (CI) in the univariate analysis and the significant prognostic factors were enrolled in multivariate analysis to confirm independent prognostic factors. Overall survival rates were evaluated using Kaplan-Meier curves with the Log-rank test. Univariable and multivariable analyses were carried out by applying the Cox proportional hazards regression model. All the experiments have been performed at least three times. All statistical analyses were performed using SPSS software (version 22.0).

Further details of the materials and methods used in this study can be found in the online supplementary files.

## Results

### Mutational landscape established by targeted DNA sequencing

**Figure 1:**
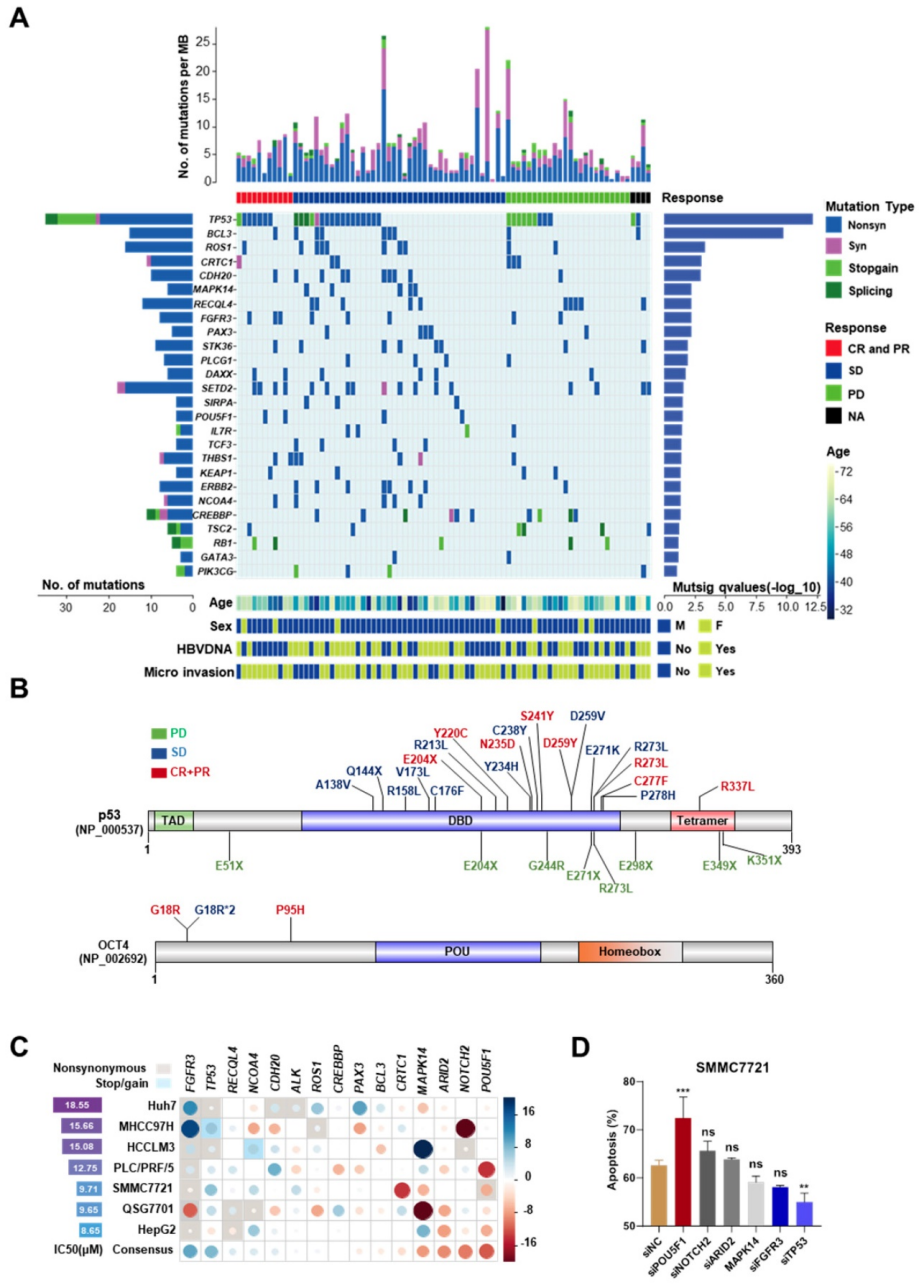
** The mutation landscape of cancer-related genes for advanced HCC and mutations associated with differential responses to sorafenib. (A)** The top panel shows individual tumor mutations rates. The second panel indicates clinical responses after sorafenib treatment. The middle panel shows genes with statistically significant levels of somatic point mutations (MutSigCV, q-value < 0.1). The bottom panel shows the age, sex, HBV-DNA, and microvascular invasion. The left panel shows gene mutation rates and the right panel shows gene mutation significant levels (as the log_10_-transformed p-value). **(B)** The nonsynonymous mutations (Frequency > 20%) in *TP53* and *OCT4* and their corresponding sorafenib responses in HCC patients. Font colors of mutations indicate different responses: green for progressive disease (PD), blue for stable disease (SD), red for complete response or partial response (CR+PR). Sites with asterisk and numbers indicated the occurrence number of the mutation. **(C)** The gene-mediated sorafenib responses by knockdown experiments. The left panel shows the IC50 for each cell line without siRNA knockdown. The circle in the matrix indicates the changes of IC_50_ in the corresponding cell line (row) after the knockdown of the corresponding gene (column). The circle with a larger radius means stronger change (measured as Z score) and the color indicates the direction of the change (blue/red for reduced/increased sensitivity after the knockdown). The background rectangle with shadow indicates that the gene in the cell line is mutated. The last line of the matrix shows the mean effect across all the tested cell lines for each gene. **(D)** Cell early and late apoptosis rate was analyzed using Flow cytometry in SMMC7721 cells treated with the indicated siRNAs along with 10uM sorafenib or DMSO. ***P<0.001; ** P <0.01; ns, not significantly, as compared to the negative control cells.

To avoid the contamination from normal hepatocytes and other non-hepatocyte components, Laser capture microdissection (LCM) was used to collect tumor and adjacent normal tissues from the first surgery of 119 HCC patients, who relapsed after the surgery and were not suitable for the second operation. All these 119 patients received mono sorafenib therapy or systemic therapy containing sorafenib; according to NCCN guidelines for advanced hepatobiliary cancers (See in Figure S1A-B and patient information in Table S1). The collected specimens were analyzed by targeted exome sequencing of 440 cancer genes (409 from Ion AmpliSeq^TM^ Comprehensive Cancer Panel and 31 custom-designed HCC-related genes, Table S3). After the default quality control and a modest filter (coverage > 150 and broadness > 60% for the targeted regions), 86 paired samples were left. Among them, 77 pairs were sequenced by Ion Proton^TM^ Systems (the median coverage ~1100×) and the other 9 were sequenced by Ion PGM^TM^ Sequencer (~300×). Additionally, 6 (4 with >1000 and 2 with >200 somatic mutations) of 86 samples were further excluded due to the abnormal high mutation rates. In the remaining 80 samples, 1,050 somatic mutations were identified, including 310 synonymous, 591 nonsynonymous, 48 stop-gain and 22 splicing mutations (Figure 1A). The average number of somatic mutations per tumor was 12.7 (6.8 per Mb), and the median was 11 (5.9 per Mb). The list of the identified somatic mutations and their basic statistics are provided in Tables S4-S8.

### Roles of highly mutated genes in cellular processes

Twenty-six significantly mutated genes (driver genes) were identified by MutSigCV (q-value < 0.1, Table S9) (Figure 1A). Four of them (*TP53, RB1, KEAP1, TSC2*) have been recurrently reported in previous studies. These twenty-six genes are strongly associated with cell cycle and cell proliferation, including* TP53* (non-silent mutation rate 45%, 34/80), *BCL3* (11.25%, 9/80), *FGFR3* (10%, 8/80),* MAPK14* (6.25%, 5/80), and* RB1* (6.25%, 5/80). *TP53* was the most mutated gene, including 3 splicing site mutations, 9 stop-gain mutations, and 22 nonsynonymous exonic mutations (MutSigCV q=7.37 × 10^-13^). Several genes encoding transcription factors, e.g., *PAX3* (6.25%, 5/80) and *OCT4* (also known as *POU5F1*, 5%, 4/80), were identified. OCT4 is a key factor for embryonic development and stem cell pluripotency 13. *PAX3* is also a regulator of fetal development 14. Chromatin modifiers play important roles in HCCs. Several highly mutated chromatin regulators reported in previous studies, such as *ARID1A* (nonsilent/silent mutations in 5/1 tumors) and *ARID2* (nonsilent/silent mutations in 5/3 tumors) were also identified, although they did not reach the threshold of statistical significance. We also identified several novel epigenetic regulator drivers, including *CREBBP*, *DAXX*, and *SETD2*. High frequent mutations in *CREBBP* have been reported previously in other cancers 15, 16.* DAXX* mutations are associated with chromatin instability and abnormal telomere maintenance 17. SETD2 is an important regulator of H3K36 methylation and therefore, affects genome accessibility and stability 18. It is interestingly found that *CTNNB1* mutations were rare in our cohort (the median coverage of *CTNNB1* region was ~1200×). A similar observation of the paucity of *CTNNB1* mutations has been made in another Chinese cohort 19.

### Mutations associated with differential response to sorafenib

Then, the relationships between mutation patterns and responses to sorafenib were analyzed. For drug responses, complete response (CR), partial response (PR), stable disease (SD), and progressive disease were assessed according to mRECIST 10. The CR and PR groups were combined as CR+PR group in this study. Four tumors without clinical response evidence were excluded from this analysis. First, we examined the relationships between the responses to sorafenib and tumor mutation burden. The six hypermutated tumors were not enriched in any response group (*P* > 0.2, Fisher's exact test). Mutations in three genes,* ALK*,* OCT4,* and *ARID2*, were found moderately associated with the responses (*P* < 0.1, Table S10) by single-gene analysis. *OCT4* mutations indicated good responses. All four *OCT4* mutations were located at the N-terminal rather than its POU homeobox domain. Three of them were recurrent mutations at c.G52C (this mutation was also found in HCC cell line 7721) (Figure 1B). Although the overall non-silent mutations of *TP53* were not significantly associated with the response to sorafenib, we observed that *TP53* stop-gain mutations were highly enriched in the PD group (*P* = 1.92 × 10^-16^) (Figure 1B).

Then, knockdown experiments were implemented to validate the effects of *OCT4* and *TP53* mutations on the sensitivity to sorafenib in HCC cell lines. In addition, two other genes with moderate associations (*ALK* and* ARID2*), and another 11 significantly mutated genes (*BCL3*, *FGFR3*, *CDH20*, *CRTC1*, *NOTCH2*, *ROS1*, *MAPK14*, *PAX3*, *RECQL4*,* CREBBP*, and *NCOA4*) were included for the experiments (the description of the mutations and experimental details can be found in Figure S2-S4 and Table S11-S12). *OCT4* knockdown strongly improved the inhibition of cell growth by sorafenib in most studied cell lines, whereas *TP53* knockdown increased the resistance to the drug (Figure 1C). Inconsistent with a previous study 20, no significant effects of *MAPK14* was found after sorafenib treatment. Both flow cytometry analysis and western blot experiments demonstrated that siRNA-induced knockdown of *TP53* significantly attenuated sorafenib-induced apoptosis and cell death in SMMC7721, whereas knockdown of *OCT4* showed the opposite effect (Figure 1D, Figure S5A-B).

### Stop-gain mutations within* Tp53* promotes sorafenib resistance

**Figure 2:**
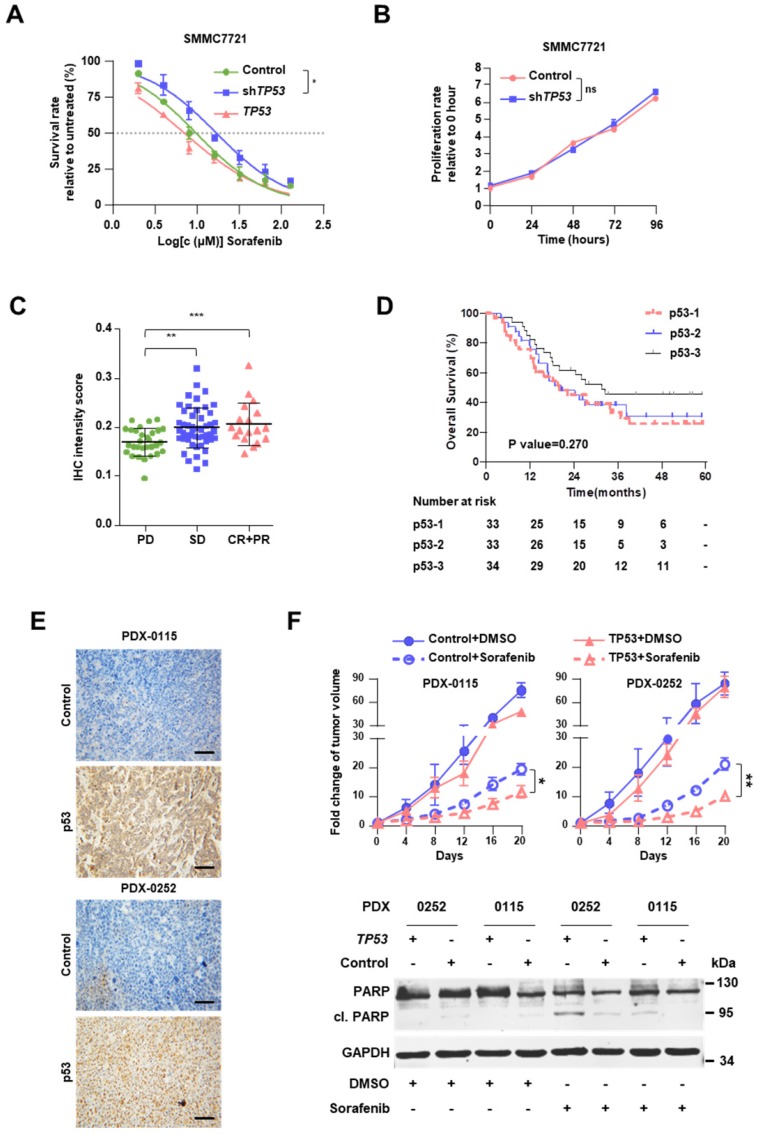
** Stop-gain *TP53* mutation promotes resistance to sorafenib. (A)** Dose-response curves for sorafenib. SMMC7721 cells harboring *GFP* (Control)*,* sh*TP53-* or *TP53*-vehicle were treated with sorafenib at the indicated concentrations. Viable cells were measured after 48 h of treatment and plotted relative to untreated control cells (mean ± s.d., n = 3 for each concentration). The curves were fitted using a nonlinear regression model with a sigmoidal dose-response.** (B)** The proliferation rates of SMMC7721 cells with Control*/*sh*TP53*-vehicle were evaluated by CCK8 assay. **(C)** The IHC intensity scores of p53 were analyzed in FFPE specimens in different response groups. **(D)** Kaplan-Meier analysis of the correlation between p53 expression levels and OS in HCC patients with sorafenib therapy. **(E)** Representative p53 staining in* TP53*- or *GFP*-lentivirus treated PDXs. **(F)** Male NOD.Cg-Prkdc^scid^ Il2rg^tm1Wjl^/SzJ mice (n=6) were subcutaneously injected with engraftments of PDXs, then Control/*TP53*-lentivirus and sorafenib were treated, the tumor volumes of engraftments of PDXs were evaluated for 20 days (top). Western immunoblot analysis for PARP and cleavage PRAP in sorafenib-treated PDX cells lentivirally transduced with Control/*TP53* (bottom)*.* ***P<0.001, ** P <0.01, * P <0.05.

Knockdown of *TP53* by infection with shRNA-carrying lentivirus (sh*TP53*) promoted resistance to sorafenib of SMMC7721 cells (Figure 2A, Figure S5C-D), but had no impact on cell proliferation (Figure 2B). In addition, overexpression of *TP53* improved the sensitivity to sorafenib, although the result did not reach statistical significance (Figure 2A). Sorafenib IC_50_ increased 1.7-fold after sh*TP53* treatment (16.99μM vs. 9.83μM) but decreased by 27.8% upon *TP53* overexpression (7.10μM vs. 9.83μM).

Immunohistochemical staining clearly showed that the level of p53 is positively associated with the clinical responses (Figure 2C, Figure S6A and Table S13). It should be noted that in our cohort p53 expression was not found as a significant prognostic indicator, the HR was 0.790 (95% CI, 0.586-1.064; P=0.119), suggesting further study with larger sample size is warranted to clarify its contribution for sorafenib response. As expected, tumor size > 5 cm and vascular invasion were also found to be significant and independent prognostic factors for the OS (Table 1). In the Kaplan-Meier estimation, the OS of the patients with high expression of p53 revealed to be higher compared to those with low or moderate expressions without statistical significance (1-year OS, 85.3% versus 75.8% and 78.8%; P=0.270; Figure 2D).

Meanwhile, Kaplan-Meier estimation showed patients with *TP53* stop gain have a poor prognosis (P=0.048, Figure S6B), compared with other *TP53* mutations. To assess the *in vivo* impact of exogenous p53 on the response to sorafenib, we employed PDX models in NOD.Cg-Prkdc^scid^ Il2rg^tm1Wjl^/SzJ mice (Figure S6C). PDXs demonstrated distinct *TP53* mutations, namely stop-gain mutation in PDX-0115 (*TP53*-NM_000546:exon8:c.G892T:p.E298X) and missense mutation in PDX-0252 (*TP53*-NM_000546:exon8:c.G818T:p.R273L). These two mutations were also detected in the sequenced specimens; they were associated with diminished protein expression of p53 in PDXs and progressive disease in HCC patients (Figure 2E). In both PDX-0115 and PDX-0252 models, lentivirus-induced overexpression of* TP53* resulted in enhanced cell apoptosis upon sorafenib treatment, which meant that restoration of *TP53* expression level significantly augmented the response to sorafenib (Figure 2F).

### *OCT4* mutation (c. G52C) sensitizes HCCs to sorafenib treatment

**Figure 3:**
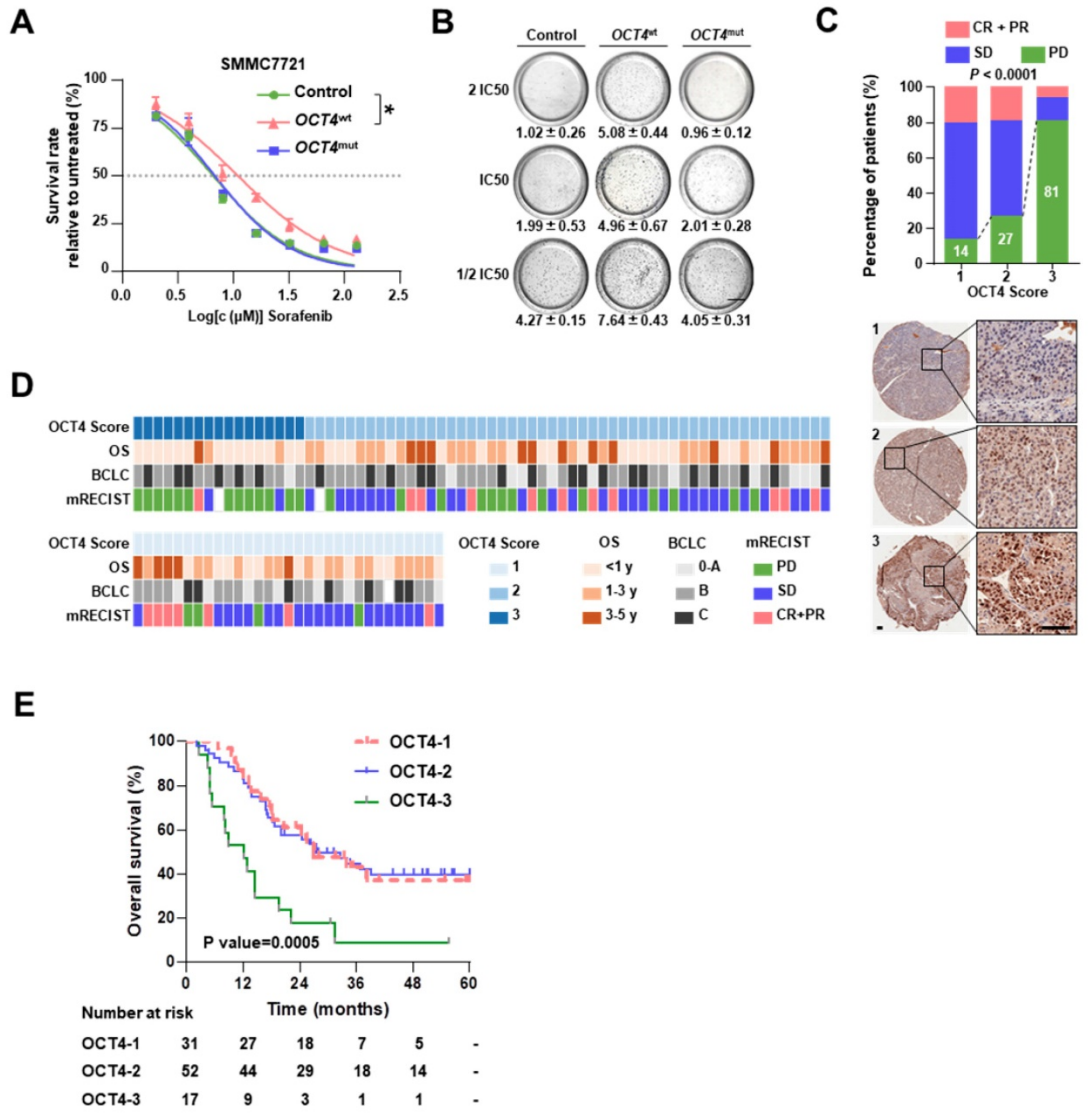
***OCT4* mutation sensitizes HCC to sorafenib. (A)** Dose-response curves for sorafenib. SMMC7721 cells transfected by *GFP* (Control), *OCT4*^wt^ or *OCT4*^mut^ (c.G52C) were treated with sorafenib at the indicated concentrations. Viable cells were measured after 48 h of treatment and plotted relative to untreated control cells (mean ± s.d., n = 3 for each concentration, *P<0.05). **(B)** Soft agar colony assay of Control-, *OCT4*^wt^ - and *OCT4*^mut^ -SMMC7721 cells treated with sorafenib at the indicated concentrations. Colonies (mean±s.d, n=3) 50 μm were counted using a microscope 21 days later. Scale bar, 800 μm.** (C)** The drug responses in different OCT4 expression sub-groups (measure by IHC score). Representative IHC staining for OCT4 was shown below. **(D)** The matrix shows the OCT4 IHC scores and the OS, BCLC stages and sorafenib responses of treated patients.** (E)** Kaplan-Meier estimation of OS according to the expression of OCT4.

As shown in Figure 1B, *OCT4* c.G52C mutation may affect sorafenib sensitivity. The *OCT4* acts as a key transcription factor to participate in tumorigenicity and drug-resistance of HCC 21.To explore the mechanism of *OCT4* mediated sorafenib response, we firstly examined the protein expression of OCT4 in nine cell lines (Figure S7A). Among the cell lines with low OCT4 levels, SMMC7721 cells (harboring the c.G52C mutation) and MHCC97H cells (without the c.G52C mutation) were separately infected with lentiviruses harboring wild-type and c.G52C-mutant *OCT4* (denoted as 7721-*OCT4*^wt^ and 7721-*OCT4*^mut^, respectively). *OCT4*^wt^ cells showed relatively higher resistance to sorafenib, whereas *OCT4*^mut^ cells were as sensitive as cells infected with control lentivirus in both cell lines (Figure 3A and Figure S7B). The majority of *OCT4*^wt^ cells survived in the presence of 2 × IC_50_ sorafenib concentration, whereas the survival rate in *OCT4*^mut^ cells declined significantly with sorafenib at 0.5 × IC_50_ concentration (Figure 3B). On the other hand, PLC/PRF/5 cells expressed wildtype* OCT4* and higher OCT4 protein levels, showed no alteration in response to sorafenib with wild-type or c.G52C-mutant *OCT4* transfection (Figure S7C). As expected, immunohistochemical staining showed that OCT4 expression was significantly higher in the PD group than in the CR+PR group (Figure 3C and 3D). A higher protein level of OCT4 was found to be an unfavorable prognostic factor that significantly reduced the OS in both univariable analysis (HR, 1.682; 95% CI, 1.128 to 2.507; *P* = 0.010) and multivariable (HR, 1.587; 95% CI, 1.026 to 2.455; *P* = 0.038) analyses (Table 1). In the Kaplan-Meier estimation, high expression of OCT4 resulted in a significant reduction in OS (1-year OS, 47.1% versus 82.7% and 83.9%; P=0.0005; Figure 3E). Additionally, the level of AFP and larger tumor size were found significantly associated with higher expression of OCT4 (*P*<0.05; Figure S6D).

### The activation of c-Kit/Flt3-Ras/Raf/MAPK signaling is indispensable for sorafenib resistance

**Figure 4:**
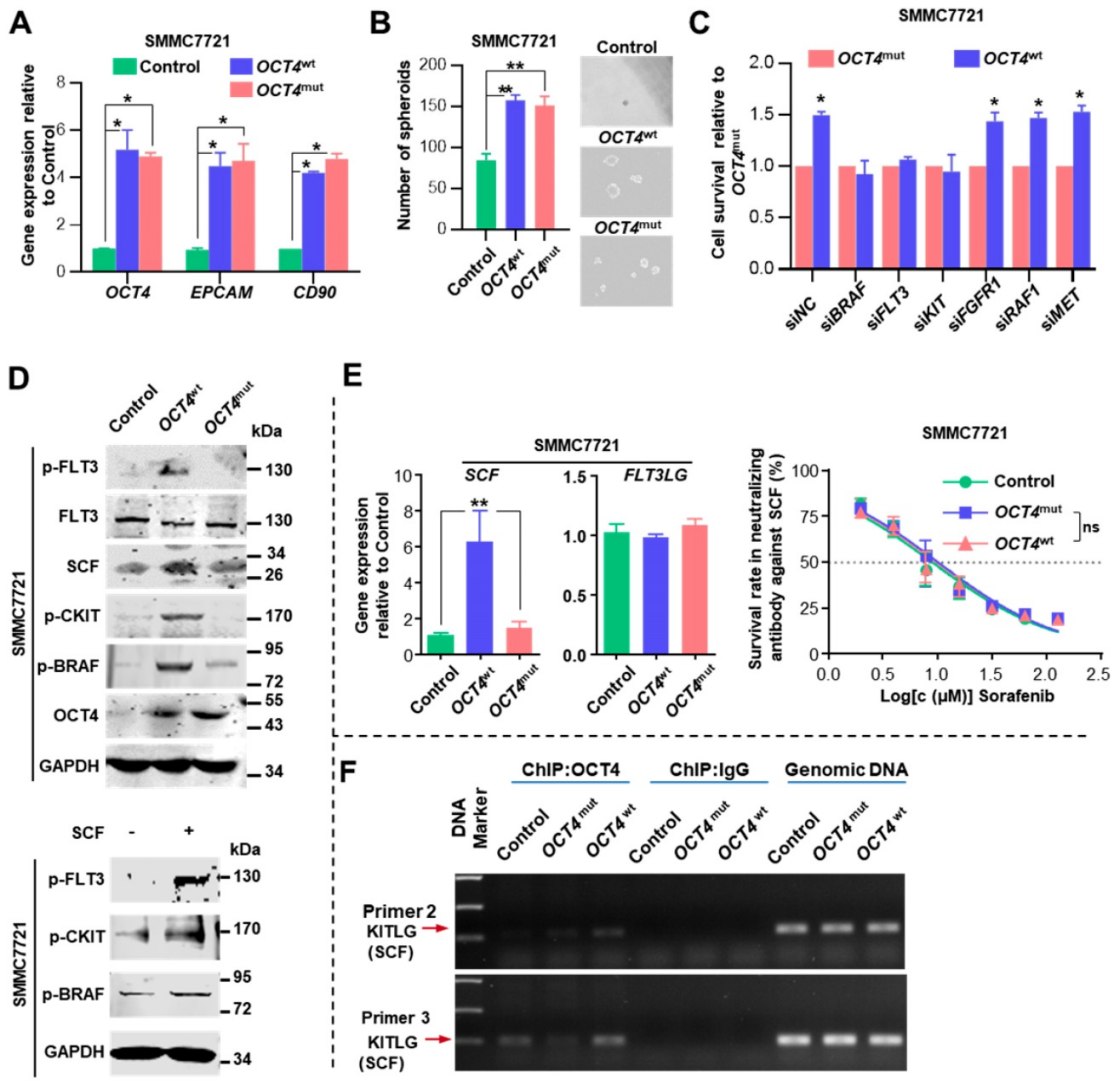
***OCT4* modulates c-KIT/FLT3 and activates RAS/RAF/MAPK signaling. (A)** The fold change of *OCT4*, *EPCAM,* and *CD90* mRNA levels was determined by qRT-PCR analysis in SMMC7721 cells treated with Control-, *OCT4*^wt^ or *OCT4*^mut^ -lentivirus. 18S was used as an internal control (mean±s.d, n=3).** (B)** Determination of tumor spheroid formation. A total of 3000 cells were seeded into low-adhesion plates and incubated for 10 days after the indicated treatment (mean±s.d, n=3). **(C)** Relative cell survival rates of *OCT4*^wt^-to *OCT4*^mut^-SMMC7721 cells with indicated siRNAs and sorafenib in IC50 of Control SMMC7721 cells for 48 (mean±s.d, n=3).** (D)**Upper panel: Levels of indicated protein determined by western blot in Control-, *OCT4*^wt^- and *OCT4*^mut^-SMMC7721 cells; Lower panel: Protein levels of p-FLT3, p-CKIT and p-BRAF determined by western blot after SMMC7721 cells stimulated with SCF (5ng/ml) for 48h. **(E)** Left panel: Detection of mRNA levels of *SCF* and *FLT3LG* in Control-, *OCT4*^wt^- and *OCT4*^mut^-SMMC7721 cells (mean±s.d, n=3); Right panel: Dose-response curves for sorafenib in the presence of neutralizing antibody against SCF. Control-, *OCT4*^wt^ - and *OCT4*^mut^ -SMMC7721 cells were treated with sorafenib at the indicated concentrations. Viable cells were measured after 48 h of treatment and plotted relative to untreated control cells (mean ± s.d., n = 3 for each concentration, **P<0.01, * P <0.05). (**F**) Chromatin immunoprecipitation from Control-, *OCT4*^wt^ - and *OCT4*^mut^ -SMMC7721 cells using OCT4 antibody. The analysis was conducted using specific primers for the promoter region of* KITLG*.

We inspected whether *OCT4*^mut^ influenced the efficacy of sorafenib by modulating tumor cell stemness. Both *OCT4*^wt^ and *OCT4*^mut^ cells expressed increased levels of *OCT4*,* EPCAM*, *CD90* genes, and exhibited enhanced spheroid formation (Figure 4A-B). Then, we examined regorafenib (another multi-kinase inhibitor approved for advanced HCCs recently) concentration-response relationship in *OCT4*^wt^ and *OCT4*^mut^-treated SMMC7721 cells. No difference of regorafenib IC_50_ value was found between cells expressing *OCT4*^wt^ and *OCT4*^mut^ (Figure S7E). Since sorafenib possess much higher IC_50_ values for FLT3 (58 nM with sorafenib) and c-Kit (68 nM with sorafenib v.s. 7 nM with regorafenib) 22, 23 in comparison with regorafenib, we wondered whether *OCT4*^mut^-induced cell sensitivity for sorafenib might result from the differential activity on FLT3 or c-Kit signal upon sorafenib or regorafenib treatment. Specifically, as shown in Figure 4C, suppressing FLT3 and c-KIT expression could partially overcome *OCT4*^wt^-induced resistance to sorafenib, whereas down-regulating the expression of BRAF, the downstream target of FLT3 and KIT 24, totally reversed the impact of *OCT4*^wt^. Western blot analysis also revealed an increased level of phosphorylated FLT3, c-KIT, and BRAF in SMMC7721 (*OCT4*^wt^) cells rather than *OCT4*^mut^ cells (Figure 4D, upper panel). Given that BRAF is a component of RAS/RAF/MAPK signaling, these results indicate that *OCT4*^wt^ might reactivate the RAS/RAF/MAPK pathway through c-KIT and FLT3 and contribute to the resistance to sorafenib.

Relative mRNA expression of stem cell factor (*SCF*, *KITLG* or c-KIT ligand), was substantially elevated in *OCT4*^wt^ cells upon sorafenib treatment, whereas the expression of *FLT3LG*, a ligand of FLT3, was unaltered (Figure 4E, left panel). Surprisingly, we observed that SCF could induce the phosphorylation of both FLT3 and c-KIT, indicating that the cross-activation of SCF upon c-KIT and FLT3 receptors (Figure 4D, lower panel). Sorafenib exhibited full efficacy in both *OCT4*^wt^ and *OCT4*^mut^ SMMC7721 cells when they were treated with the neutralizing antibody against SCF (Figure 4E, right panel). Last but not least, chromatin immunoprecipitation (ChIP) showed that more OCT4 was recruited to the predicted promoter regions of the KITLG (the gene encodes stem cell factor) in *OCT4*^wt^ SMMC7721 cells than in control- or *OCT4*^mut^ SMMC7721 cells (Figure 4F and Figure S7D). Taken together, these results suggested that in* OCT4*^wt^ overexpressing HCC, sorafenib-induced SCF expression and simultaneous activation of c-KIT and FLT3 signaling is indispensable for *OCT4*^wt^ mediated sorafenib resistance.

### Combined treatment with sorafenib and tyrosine kinase inhibitors (TKIs) improves therapeutic response

**Figure 5:**
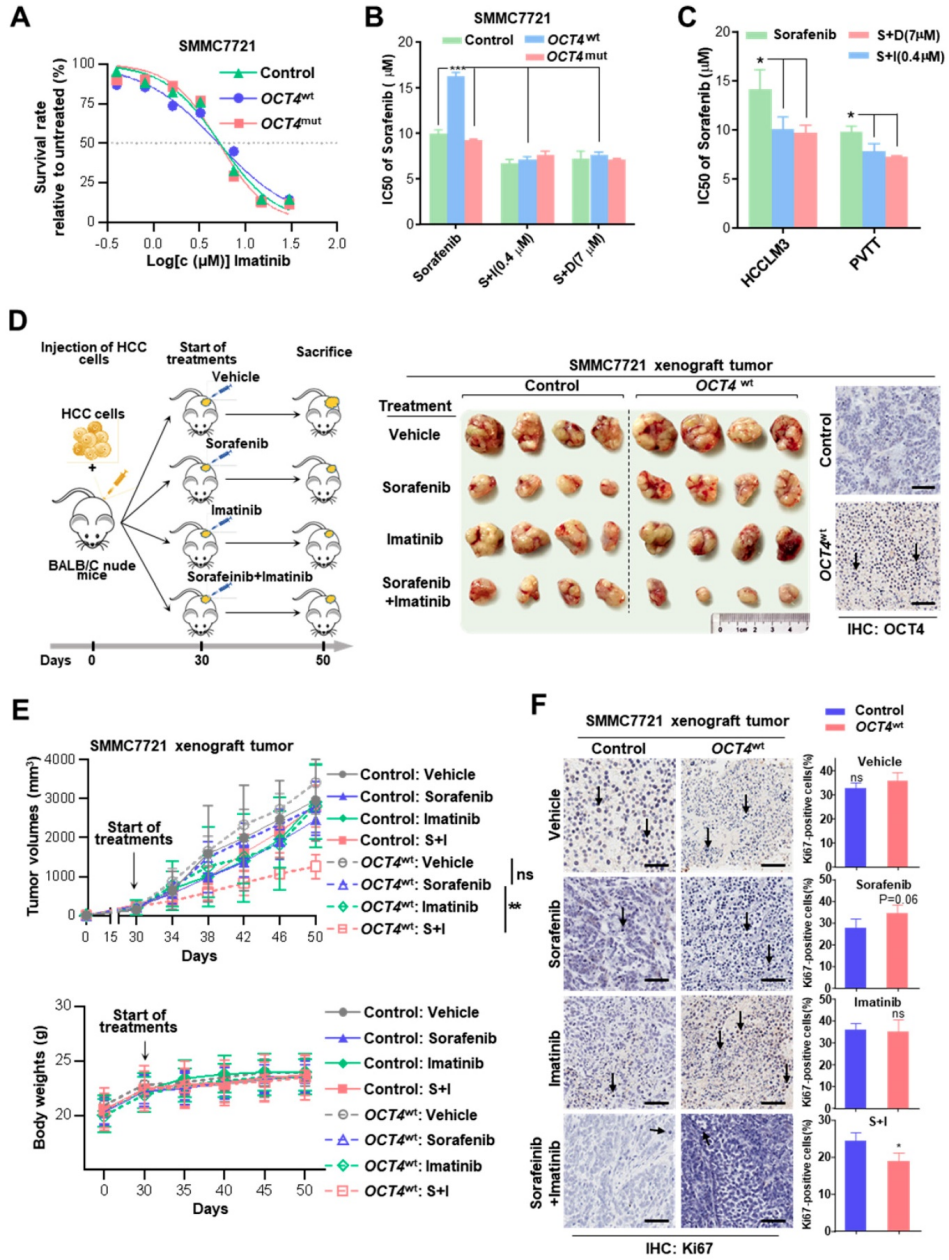
** Combined treatment with sorafenib and c-KIT tyrosine kinase inhibitors improves drug's impact. (A)** Dose-response curves for imatinib. Control-, *OCT4*^wt^ - and *OCT4*^mut^ -SMMC7721 cells were treated with imatinib at the indicated concentrations. Viable cells were measured after 48 h of treatment and plotted relative to untreated control cells (mean ± s.d., n = 3 for each concentration). **(B, C)** Control-, *OCT4*^wt^ - and *OCT4*^mut^ -SMMC7721 cells **(B)** and HCC-LM3, PVTT cell lines **(C)** were treated by sorafenib alone or combined with 0.4μM imatinib (S+I) and 7μM dovitinib (S+D), respectively. Viable cells were measured after 48 h of treatment and plotted relative to untreated control cells. Dose-response curves were fitted and IC_50_s of each treatment were calculated.** (D)** Left: Schematic representation of establishing HCC xenograft tumor burden in mice with mono sorafenib or combined sorafenib and imatinib treatment; Right: Intrahepatic tumor burden of nude mice 20 days after Control- and *OCT4*^wt^-SMMC7721 cells injection and treatment with sorafenib, imatinib, combinations thereof and vehicle (n = 4). **(E)** Tumor volume (upper panel) and weight changes (lower panel) in Control-, *OCT4*^wt^ - and *OCT4*^mut^ -SMMC7721 cells after vehicle-, sorafenib- and sorafenib combined imatinib-treatment (mean ± s.d., n = 4 for each condition).** (F)** Left: Representative Immunohistochemistry images of Ki-67 staining in xenografts generated from subcutaneous transplantation with indicated treatment; Right: Quantification of Ki67-positive cells in corresponding xenografts in the left. (mean ± s.d., n = 3,statistical significance calculated using Student's t-test, scale bars: 100 µm, *** *P* <0.001, ** P <0.01, * P <0.05).

Due to the cross-activation of c-KIT/FLT3 signaling, the efficacy of the combined treatment with imatinib (a selective tyrosine kinase inhibitor of c-KIT and platelet-derived growth factor receptor) or dovitinib (TKI-258, a multi-targeted receptor tyrosine kinase inhibitor for FLT3/c-KIT) was evaluated to verify whether it can improve the sensitivity of sorafenib therapy. As shown in Figure 5A and Figure S7F, no difference was observed for the cell survival among control, *OCT4*^wt^ and *OCT4*^mut^ SMMC7721 cells in the presence of either of two TKIs. Remarkably, *OCT4*^wt^ cells had a better response to the treatment of sorafenib and c-KIT TKIs than sorafenib alone (Figure 5B and Figure S7G).

The effects of combined sorafenib and c-KIT TKIs treatment were further examined in *OCT4*^wt^ cell lines (HCCLM3 and PVTT). As expected, the combination treatment achieved much better responses for both HCCLM3 and PVTT cells than sorafenib only (Figure 5C). Moreover, the efficacy of combined sorafenib and imatinib treatment was evaluated in xenografts inoculated with lenti-*OCT4*^wt^-infected SMMC7721 cells or lenti-NC-infected cells. *OCT4*^wt^ xenografts showed a limited response to monotherapy with either sorafenib or imatinib, whereas the combination of two drugs resulted in significant tumor regression and the absence of weight loss in mice (Figure 5D-E). Notably, markedly decreased expression of the cell proliferation marker Ki-67 in HCC cells was observed in xenografts treated with combined sorafenib and imatinib (Figure 5F).

## Discussion

**Figure 6:**
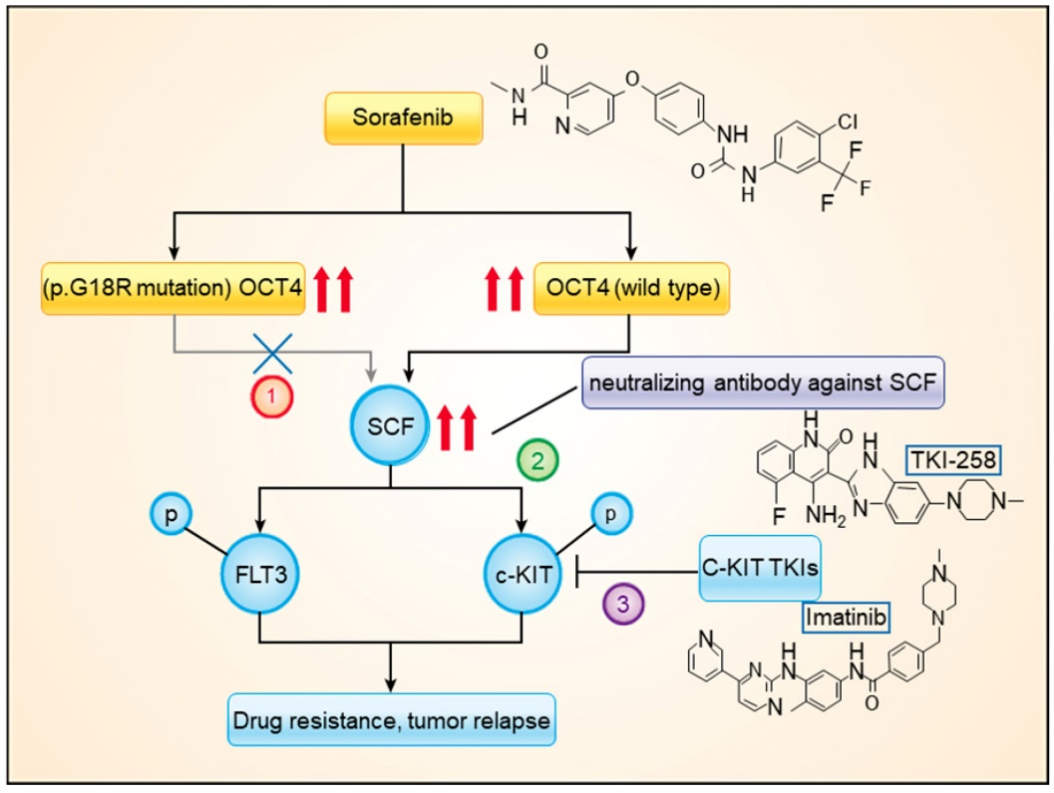
** A model showing the mechanism of *OCT4*^wt^ rendering sorafenib resistance.** Combined treatment with sorafenib and imatinib or neutralizing antibody against SCF blocked the activation of c-KIT by *OCT4*^wt^.

In the present study, by using high-throughput sequencing, we explored the genetic features in HCC tissue samples that were associated with the response to sorafenib treatment. Among the frequently mutated genes, the stop-gain mutation of *TP53* and the c.G52C mutation of *OCT4* (p.G18R of OCT4) were found to be associated with sorafenib responses. The *OCT4*^wt^ was found to modulate expression levels of c-KIT and FLT3, which were simultaneously activated by SCF, upregulating RAS/RAF/MAPK signaling. The combined treatment with sorafenib and imatinib or dovitinib had significantly higher efficacy than that of sorafenib mono treatment (Figure 6).

A phase III study of pembrolizumab, as a second-line agent after sorafenib therapy recently reported negative results 25. At present, multi-kinase inhibitors, which are represented by sorafenib, still serve as the first-line treatment for patients with unresectable HCC 26. However, this promising systemic treatment provides only limited survival benefits and has a low response rate, indicating that there is a great need to study the mechanism of the drug resistance to improve survival outcomes of patients with HCC. To the best of our knowledge, this study is the first large-scale clinical genomic study of primary resistance to sorafenib in aHCC. Unexpectedly, unlike the general consensus that the efficacy of EGFR tyrosine kinase inhibitors and anti-EGFR antibodies should be affected first and foremost by EGFR mutations, the efficacy of sorafenib was altered by mutations in other genes rather in its primary targets.

In accordance with previous studies that reported 58% *TP53* mutation rate in HCC patients 27 and *TP53* mutation were significant prognostic factors associated with shorter survival 28, our data showed that 37.9% of HCC cases had *TP53* mutations, which ranked *TP53* as the most mutated gene in our cohort. There are lots of reports on the role of *TP53* genetic alterations in HCC: The R273L mutation is a dominant-negative mutation that contains a point mutation in the DNA-binding domain and is commonly found in various types of cancers 29; Inactivating mutations of *TP53* were one of the main potential drivers and enriched in HBV-related HCC 30. Besides, *TP53* mutation also resulted in the downregulation of the immune response and doxorubicin resistance in HCC 31, 32. We initially found the TP53 stopgain mutation in HCC as a mutation type conferring resistance to sorafenib. The stop-gain variant E298X, which diminished p53 expression, also demonstrated a significant impact on the sensitivity to sorafenib. Since frameshift mutation also leads to TP53 loss-of-function, we attempted to examine whether stopgain mutation and frameshift mutation would make any difference in drug response by checking the ICGC database. As expected, both stopgain and frameshift cases showed worse survival rates in comparison with missense cases. When combining cases with frameshift and stopgain together, a more obvious difference between missense and frameshift+stopgain groups was observed (data not shown). Further studies with a larger cohort with either TP53 mutation cases or drug response information should be warranted. In our survival analysis, patients with higher p53 expression showed better OS albeit, without statistical significance, this correlation should be further determined within a larger cohort. Although it has been reported that over-expression of p53 resulted in poor survival in HCC 33, the inconsistency in the present study might be due to the different inclusion criteria and clinical management with sorafenib for HCC patients nowadays. Future studies will be necessary to find ways to overcome the resistance by combining sorafenib administration with the restoration of wild-type *TP53* activity. We believe that the suppression of the inhibitory impact of *TP53* loss-of-function mutations may increase the therapeutic efficacy of sorafenib for aHCC patients.

Although *OCT4* has been suggested to play a crucial role in HCC 34, its mutations have not been explored for drug resistance. Higher expression of *OCT4* was observed in sorafenib-resistant cells compared with that in their parental counterparts at the mRNA and protein levels 35. Our data functionally identified *OCT4*^wt^ as a target whose upregulation confers strong resistance to sorafenib treatment. It has been reported that knockdown of KITLG with siRNA and inhibition of SCF signaling by tyrosine kinase inhibitors like sorafenib could enhance anti-tumor reactivity, tumor regression and prolonged survival in murine models of colon and Lewis lung carcinoma^36^, ^37^, and we firstly identified the KITLG gene was a novel OCT4-responsive gene that was upregulated upon wild-type OCT4 overexpression. Furthermore, the increased level of *OCT4*^wt^ was associated with phosphorylation of c-KIT and FLT3 through the release of SCF and consequently reactivated the RAS/RAF/MAPK cascade. As our results indicated, the activation of c-KIT by *OCT4*^wt^ predicted poor response to sorafenib treatment. However, *OCT4*^mut^ did not exert such effects. These results provided a clue for the mechanisms of sensitivity to sorafenib in certain populations and suggested a new drug combination approach. The lack of toxicity is particularly important for the treatment of patients with advanced HCC with impaired liver function 38, 39. Thus, it is noteworthy that imatinib may (without additional toxicity) potentially restore the sensitivity to sorafenib in cancers that have become resistant 40. In addition, results of a recent phase 2 study showed another c-KIT/FLT3 inhibitor dovitinib, also known as TKI-258, did not appear to have improved activity over sorafenib in patients with advanced HCC 41, our data suggested that future trials can be designed to test the therapeutic efficacy of a combined treatment with sorafenib and imatinib or dovitinib in patients with high expression levels of *OCT4*^wt^ and c-KIT.

Analysis of clinical data suggested that both p53 and OCT4 were independent prognostic factors, of which p53 expression was associated with favorable survival outcomes, whereas expression of OCT4 indicated unfavorable prognosis. More importantly, in comparison with G52C mutation, our study here implied that the expression of OCT4 seems to be a more practical biomarker for drug response prediction. Patients with a higher expression level of OCT4 might significantly benefit from the combined treatment with sorafenib and c-KIT TKIs than sorafenib mono-treatment.

Because mutations of certain genes serve as negative factors that enhance sorafenib resistance, should sorafenib treatment be replaced with second-line therapies in HCC patients with such mutations? With the recent advances in the development of second-line therapeutic agents, such as regorafenib, precise selection of patients for whom sorafenib treatment would be beneficial becomes a major issue 42. Future trials exploring relevant stratification of patients and identification of those who will and who will not likely obtain survival benefit from sorafenib treatment are necessary.

Although our study was performed on the largest cohort of aHCC patients in which sensitivity to sorafenib responses was examined in parallel with genomic characterization, there still are some limitations. Our study was consistent with the results of other studies from Eastern countries: all involved patients were from mainland China, where HBV infection is endemic. The adverse events of sorafenib were not collected because of the retrospective nature of the study. Similarly, the adverse events during the combined treatment with sorafenib and imatinib could not be evaluated. Despite these limitations, our study uncovered several molecular markers of primary sorafenib resistance, including *TP53, OCT4* mutations, and *OCT4* overexpression, which have not been reported previously. The combination of clinical genomic analysis and functional study will provide informative clues for future trials. We highly suggest future large-scale randomized controlled trials to assess the impact on sorafenib responses in patients with *TP53* stop-gain mutations. And also, combined therapy with sorafenib and other tyrosine kinase inhibitors may be recommended in patients with certain genetic alterations who failed or progressed on sorafenib treatment.

## Supplementary Material

Supplementary materials and methods, figures.

Supplementary tables.

## Figures and Tables

**Table 1:** Univariate and Multivariate Analyses (Cox regression)

Characteristic	Univariate analysis		Multivariate analysis	
	HR (95% CI)	*P* value	HR (95% CI)	*P* value
**p53**	0.790 (0.586-1.064)	**0.119**	NA	NA
**OCT4**	1.682 (1.128-2.507)	**0.010**	1.587 (1.026-2.455)	**0.038**
**Tumor size > 5 cm**	1.818 (1.029-3.211)	**0.037**	2.483 (1.226-5.028)	**0.012**
**Macrovascular Invasion**	2.083 (1.210-3.586)	**0.007**	2.146 (1.231-3.742)	**0.007**

Abbreviations: HR, hazard ratio; CI, confidence interval. A value of *P*<0.05 was considered to be significant.

## Abbreviations

HCC: hepatocellular carcinoma
HBV: hepatitis B virus
BCLC: Barcelona Clinic Liver Cancer
OS: overall survival
PDX: patient-derived xenograft
FFPE: formalin-fixed paraffin-embedded
LCM: laser capture microdissection
mRECIST: modified Response Evaluation Criteria in Solid Tumors
qRT-PCR: quantitative reverse-transcript polymerase chain reaction
