# Supplementary material for: Targeted sequencing reveals the mutational landscape responsible for sorafenib therapy in advanced hepatocellular carcinoma
Source: Theranostics. 2020 Apr 6;10(12):5384–97. doi: 10.7150/thno.41616 (PMC7196302; doi:10.7150/thno.41616)

Supplementary Materials:

Targeted sequencing reveals the mutational landscape responsible for sorafenib therapy in advanced hepatocellular carcinoma

Jing Tang,[1,2,#] Cheng-Jun Sui,[3#] Dong-Fang Wang,[4,#] Xin-Yuan Lu,[5,#] Gui-Juan Luo,[1] Qing Zhao,[6] Qiu-Yu Lian,[4]Seogsong Jeong,[1,7] Xi-Meng Lin,[1] Yan-Jing Zhu,[1] Bo Zheng,[1] Rui Wu,[8] Qing Wang,[1] Xiao-Long Liu,[9] Jing-Feng Liu,[9] Qiang Xia,[7] Gang Wu,[2] Jin Gu,[4,]* Hong-Yang Wang,[1,11,12,]* and Lei Chen[1,10,]*

Table of contents

Supplementary Materials and Methods.. **2**

Supplementary Figure Legend **8**

Reference.. **12**

Supplementary Figure S1-S7 .... **13**

Supplementary Materials & Methods

Cell Lines and Lentivirus

Cell lines used in this study were QSG-7701, PLC/PRF/5, SMMC-7721, MHCC-97H, Huh7, HepaG2, and HCC-LM3 purchased from Cell Bank of Type Culture Collection of Chinese Academy of Sciences. STR Profiling for SMMC-7721 was supplied in the supplementary materials, as most of the experiments relied on this cell line. Cells were maintained at 37 °C in an atmosphere containing 5% CO2 in Dulbecco's modified Eagle's medium supplemented with 10% fetal bovine serum. Lentivirus vectors containing the GFP, human TP53 gene and shTP53, wild type and mutated (c.G52C) human OCT4 gene were constructed, respectively. Lentivirus production was completed by the Genechem Company (Shanghai, China). SMMC-7721 or PDX cells were infected with the concentrated virus at a multiplicity of infection of 20 in the presence of polybrene (8 μg/mL) for 6 h. After 24 hours, the supernatant was replaced with fresh medium. Expression of proteins p53 and OCT4 in the infected cells was validated by IHC and Western blotting.

Animal experiments

All in vivo experiments were performed in accordance with the Eastern Hepatobiliary Surgery Hospital Research Ethics Committee. PDXs were harvested and dissociated into single cells following the passaging procedure described above. Tissue fragments (containing approximately 8-10 x 10^6 cells) were implanted in both flanks of 6-7 weeks old NOD. Cg-Prkdcscid Il2rgtm1Wjl/SzJ (NSG) mice (Model Animal Research Center, Nanjing University). Tumor volume was calculated as follows: [length * width^2 *π/6]. In

4 weeks post implantation, the volume of successful engraftments of PDXs achieved approximately 100 mm^3, 16 mice were randomized to treatment/control groups and intratumorally injected with TP53/Control lentivirus daily for a week, 4 mice in each group were intraperitoneally injected with sorafenib (50mg/kg) daily or DMSO control for 12 days, respectively. Tumor volume was calculated in every 4 days after sorafenib/DMSO treatment. Mice were sacrificed 21 days post sorafenib treatment, and their xenografts were excised, fixed in formalin (Sigma-Aldrich), and embedded in paraffin.

For SMMC7721 cell line affected with *OCT4*^{wt} and *GFP* lentivirus, thirty-two 6-7 weeks old nude mice (Model Animal Research Center, Nanjing University) were randomized to 8 treatment groups: 1.DMSO control + vehicle; 2. imatinib + vehicle; 3. sorafenib + vehicle; 4. imatinib + sorafenib + vehicle; 5. DMSO control + *OCT4*^{wt}; 6. imatinib+ *OCT4*^{wt}; 7. sorafenib + *OCT4*^{wt}; 8. imatinib + sorafenib + *OCT4*^{wt}. $1x10^6$ cells were seeded in 100 μl of PBS and injected subcutaneously in the single flank of each mouse. On the 30th day of post inoculation, lenti-*GFP*/*OCT4*^{wt} xenografts were respectively treated with drugs mentioned above, tumor volume was calculated in every 4 days, mice were sacrificed 20 days after treatment and their xenografts were excised, fixed in formalin, and embedded in paraffin.

Immunohistochemistry

Immunohistochemical staining was performed as described previously[1] in 100 available pairs of HCC patients who underwent sorafenib based treatment (Supplementary Table S13). Briefly, formalin-fixed paraffin-embedded sections of

tissues were cut into 4 μm-thick sections. The sections were deparaffinized and rehydrated. After antigen repair, the sections were incubated with 3% H2O2 for 15 min to block endogenous peroxidase activity. Primary rabbit polyclonal p53 antibody (dilution 1:200, Abclonal, A3185), rabbit polyclonal OCT4 antibody (dilution 1:200, Abcam, ab74538) and rabbit monoclonal Ki67 antibody (dilution 1:500, Abcam, ab16667) were incubated at 4 °C overnight. The sections were incubated with the secondary antibody anti-rabbit HRP (dilution 1:200, Abcam, ab97080) for 30 min at 37 °C. The nuclear OCT4 index (1, 2 and 3) of each HCC aggregates in tissue array was determined by counting of positively stained cells with the Aperio ImageScope v.12, positive pixel count algorithm software (Vista, CA). The intensities of p53 were also examined with Aperio ImageScope v.12. The scores of p53 were continuous by simultaneously evaluating cytoplasmic and nuclear localization of the protein. The scores were adjusted into high (p53-3), moderate (p53-2), and low (p53-1) expressions by stratifying the expressive ranges of the values obtained in tissue array. For analysis of associations between p53 and OCT4 expressions and clinicopathological characteristics, HCC patients were halved according to the median of IHC intensity.

RNA interference screen

For interfering RNA assay, the cells in the 6-well plate were transfected with small interfering RNAs (Genechem, Shanghai, China) by INTERFERin transfection reagent (Polyplus, France) according to the manufacturer's instructions. Cell viability was measured using the Cell Counting Kit-8 (CCK-8, Dojindo Laboratories) in 96-well

culture plates. Each experiment was repeated three times. Raw data from each plate were normalized and analyzed according to a previously described method[2]. Significantly enhanced resistance is set as *z*-score < -4 and p-value < 0.01 and improved sensitivity is set as *z*-score > 4 and p-value < 0.01. The summarized or consensus z-scores (the last row of Figure 1C) for each effector gene are computed as Stouffer's *z*-score, which is the sum of *z*-scores from all cell lines divided by the square root of the number of cell lines. To validate the effect on cell viability of RNA interference against the mutated genes, the gene expression levels, and siRNA-mediated knockdown was evaluated in the corresponding cell lines using quantitative PCR.

Soft agar assay

Low-temperature agarose (Sigma) dissolved in PBS was autoclaved and diluted to 0.7% with complete growth medium. One milliliter of 0.7% agar was cast into 12-well plates and allowed to cool down for 15 min at 4 °C to form the bottom agar. 20,000 cells were diluted in 1 ml of complete growth medium. Subsequently, cells were mixed at a ratio of 1:1 with 1 ml of 1.4% agar and carefully layered onto the bottom agar. The 12-well plates were left at room temperature for the agar to solidify before incubation in a standard tissue culture incubator. After 24 h, 1 ml of complete medium with the indicated concentration of sorafenib in Figure 3B was carefully added on top of the agar to prevent cells from drying out. After 21 days pictures were taken and colony size was measured. Each experiment was repeated three times.

PCR-based Sanger resequencing

Primers were designed according to the target regions of interest and were used to amplify these regions by PCR for genomic resequencing. Sanger sequencing was carried out by Shenzhen Huada Gene Sci-Tech Company (Shenzhen, China). The resulting data were screened for mutations through automated and manual steps. Each experiment was repeated three times.

Antibodies

The primary antibodies and the respective applications they were used are shown in Table S14.

DNA and RNA extraction

Captured specimens were collected into tubes by gravity. For each sample, 8 sections each with a thickness of to 10 µm (surface area of ~200 mm^2) were applied to DNA and RNA extraction. Paraffin is removed from FFPE LCM sections using a Deparaffinization Solution (Qiagen). DNA extraction is performed using the AllPrep DNA/RNA FFPE kit (Qiagen) according to the AllPrep DNA/RNA FFPE Handbook. DNA of Fresh frozen tissues was extracted using AllPrep DNA/RNA Mini Kit (Qiagen) according to the AllPrep DNA/RNA Mini Handbook. Isolated DNA and RNA were stored at -20 ℃ and -80 ℃ for further analysis, respectively.

Apoptosis assay

SMMC7721 cells were seeded at a density of 30% cells in 12-well plates. After adhesion, the cells were respectively transfected with the indicated siRNAs for 48 h, then treated with sorafenib for 48 h. Post-treatment, the cells were collected and centrifuged at 400 × g for 5 min at 4 °C. Cell apoptosis was determined via flow

cytometer (CytoFLEX; Beckman Coulter, Brea, CA, USA) using an Annexin V-FITC/PI Apoptosis detection kit (Vazyme, Jiangsu, China). The data was analyzed using CytExpert (Beckman Coulter) and the percentage of apoptotic cells was calculated and represented in a bar diagram.

Chromatin Immunoprecipitation (ChIP) Assay

We used the STORM algorithm[3] from the CREAD package to predict the potential binding sites of OCT4 in the KITLG promoter. Four high-quality matrices of OCT4 binding sites were extracted from the TRANSFAC database. And the upstream 3000 to downstream 2000 flanking region of KITLG transcription start site (TSS) is used for binding site predictions. The highest-scoring binding site predicted by STORM was used for the following ChIP experiments. The ChIP assay for analysis of OCT4 transcriptional activity was performed. Rabbit polyclonal antibody specific for OCT4 (Abcam, ab181557) was used. Specific primer pairs for the predicted binding site of KITLG promoter regions were used for investigating the binding of OCT4 to DNA. The primer sequences are shown in Table S15.

Supplementary Figure Legend

Figure S1 (A) Flowchart of the patients' inclusion and exclusion for targeted DNA sequencing. (B) Characterization of the technique based on LCM used for retrieval of either HCC or normal liver tissues from paraffin-embedded tissue sections. a and b, H&E staining; c and d, the same field of unstained tissue in panel a and b； e and f, removal of HCC and normal liver tissues. Bar, 100 μm. (C) Sanger sequence chromatograms of the detected stopgain *TP53* mutations in patients with PD. Arrow indicated the translation direction. (D) Sanger sequence chromatograms of the detected c.G52C *OCT4* mutations in 4 HCC cell lines. Arrow indicated the translation direction.

Figure S2 Distribution of mutations with a frequency over 20% and their relationships with sorafenib efficacy in the HCC samples is shown on a schematic of the proteins. Font colors of mutations indicate the responses to sorafenib. Green, progressive disease (PD); Blue, stable disease (SD); Red, complete response and partial response (CR+PR). Sites with asterisk and numbers indicated the frequency of occurrence of the mutation.

Figure S3 Effects of mutated genes on sorafenib response as determined using cell-based RNAi. (A) Flowchart of the cell viability assay screen for sorafenib sensitivity. The cells were treated with the siRNAs in 6-well culture plates for 24 h and re-seeded in 96-well culture plates, then sorafenib was added to the adherent cells at final concentrations of IC50. After 48 h, the cell viability was assayed with the Cell Counting Kit-8. The raw absorbance value readouts were normalized, and Z scores

and P values were calculated to evaluate the siRNA effects on sorafenib sensitivity. (B) Evaluation of cell viability in response to siRNA against 14 mutated genes in 7 HCC cell lines. Readout data from the cell-based RNAi screen were subjected to normalization. Two statistical parameters, the Z score, and the P-value were used to evaluate the effectiveness and reliability of each gene-targeting siRNA. The cutoffs for significance were Z score < −4, P-value < 0.05 (blue box) and Z score > 4, P-value < 0.05 (red box).

Figure S4 The efficacies of RNA interference *CDH20, MAPK14, NOTCH2, ROS1, OCT4, ALK, ARID2, CRTC1, PAX3, CREBBP, NCOA4, RECQL4, TP53, FGFR3 and BCL3* in the HCC cells were evaluated with real-time quantitative PCR. The statistical significance of the gene knockdown results was determined using a Student t-test. * P < 0.05; ** P < 0.01; *** P < 0.001. The most efficient siRNA of each gene was used for further experiments.

Figure S5 (A) Assessment of apoptosis using flow cytometry in SMMC7721 cells treated with sorafenib (10 μM) and indicated siRNAs. In the representative images, cells in the lower right (LR) and upper right (UR) quadrant represent early and late apoptosis, respectively. (B) Expression of total and cleaved PARP in SMMC7721 cells treated with the indicated siRNAs along with 10 μM sorafenib or DMSO. (C) Western immunoblot analysis for SMMC7721 cells affected with sh*TP53* lentivirus. (D) The efficacies of RNA interference in sh*TP53* SMMC7721 cells were evaluated with real-time quantitative PCR. **** P < 0.0001.

Figure S6 (A) Left: Matrix indicates the relationship between p53 IHC scores, overall survival (OS) of sorafenib-treated patients, BCLC stage and sorafenib response in each individual. PD, progressive disease; SD, stable disease; CR, complete response; PR, partial response; Right: Representative IHC staining for p53. P53 expression on HCC cells is assessed by immunohistochemistry assay and scored using Aperio ImageScope v.12 and graded according to tertile. Representative IHC staining for p53 was shown in the right pane. Scale bar, 100 μm. (B) Kaplan-Meier estimation of OS according to the mutation status of *TP53*. (C) Flowchart to evaluating the *TP53* effects on sorafenib sensitivity in PDX models. Surgically removed tumoral and normal liver tissues were harvested. Each tissue was applied to DNA ampliseq sequencing and establishment of PDX models as described in material and methods. In 4 weeks post implantation, the volume of successful engraftments of PDXs achieved approximately 100 mm^3, 16 mice were randomized to treatment/control groups and intratumorally injected with *TP53/*Control lentivirus daily for 7 days, 4 mice in each group were intraperitoneally injected with sorafenib (50mg/kg) daily or DMSO control for 12 days, respectively. Tumor volume was calculated in every 4 days after sorafenib/DMSO treatment. Mice were sacrificed 21 days post sorafenib treatment, and their xenografts were excised, using for further experiments. (D) Correlations between p53 and OCT4 expressions and clinicopathological characteristics in patients with HCC.

Figure S7 (A) Western immunoblot analysis for OCT4 in 9 HCC cell or hepatocyte lines. (B, C) Dose-response curves for sorafenib. MHCC97H (B) and PLC/PRF/5(C)

cells transfected by GFP (Control), *OCT4*^{wt} or *OCT4*^{mut} (c.G52C) were treated with sorafenib at the indicated concentrations. Viable cells were measured after 48 h of treatment and plotted relative to untreated control cells (mean ± s.d., n = 3 for each concentration, ns: not significant). (D) Upper lane: Sequence of the highest-scoring binding site predicted by STORM used for the following ChIP experiments. Primers for ChIP-PCR were noted with differently colored boxes; Lower lane: Chromatin immunoprecipitation from Control-, *OCT4*^{wt} - and *OCT4*^{mut} -SMMC7721 cells using OCT4 antibody. The analysis was conducted using specific primers for the promoter region of *KITLG*. (E) Dose-response curves for regorafenib. Control-, *OCT4*^{wt} - and *OCT4*^{mut} -SMMC7721 cells were treated with regorafenib at the indicated concentrations. Viable cells were measured after 48 h of treatment and plotted relative to untreated control cells (mean ± s.d., n = 3 for each concentration). (F) Dose-response curves for dovitinib (TKI-258) in SMMC7721 cells with indicated treatment. (G) Western blot analysis for PARP and cleavage PRAP in Control- and *OCT4*^{wt}-SMMC7721 cells after 48 h of sorafenib treatment.

Reference

1. Li L, Chen L, Hu L, Liu Y, Sun HY, Tang J, et al. Nuclear factor high-mobility group box1 mediating the activation of Toll-like receptor 4 signaling in hepatocytes in the early stage of nonalcoholic fatty liver disease in mice. Hepatology. 2011; 54: 1620-30.

2. Boutros M, Bras LP, Huber W. Analysis of cell-based RNAi screens. Genome Biol. 2006; 7: R66.

3. Schones DE, Smith AD, Zhang MQ. Statistical significance of cis-regulatory modules. BMC Bioinformatics. 2007; 8: 19.

Supplementary Figure 1

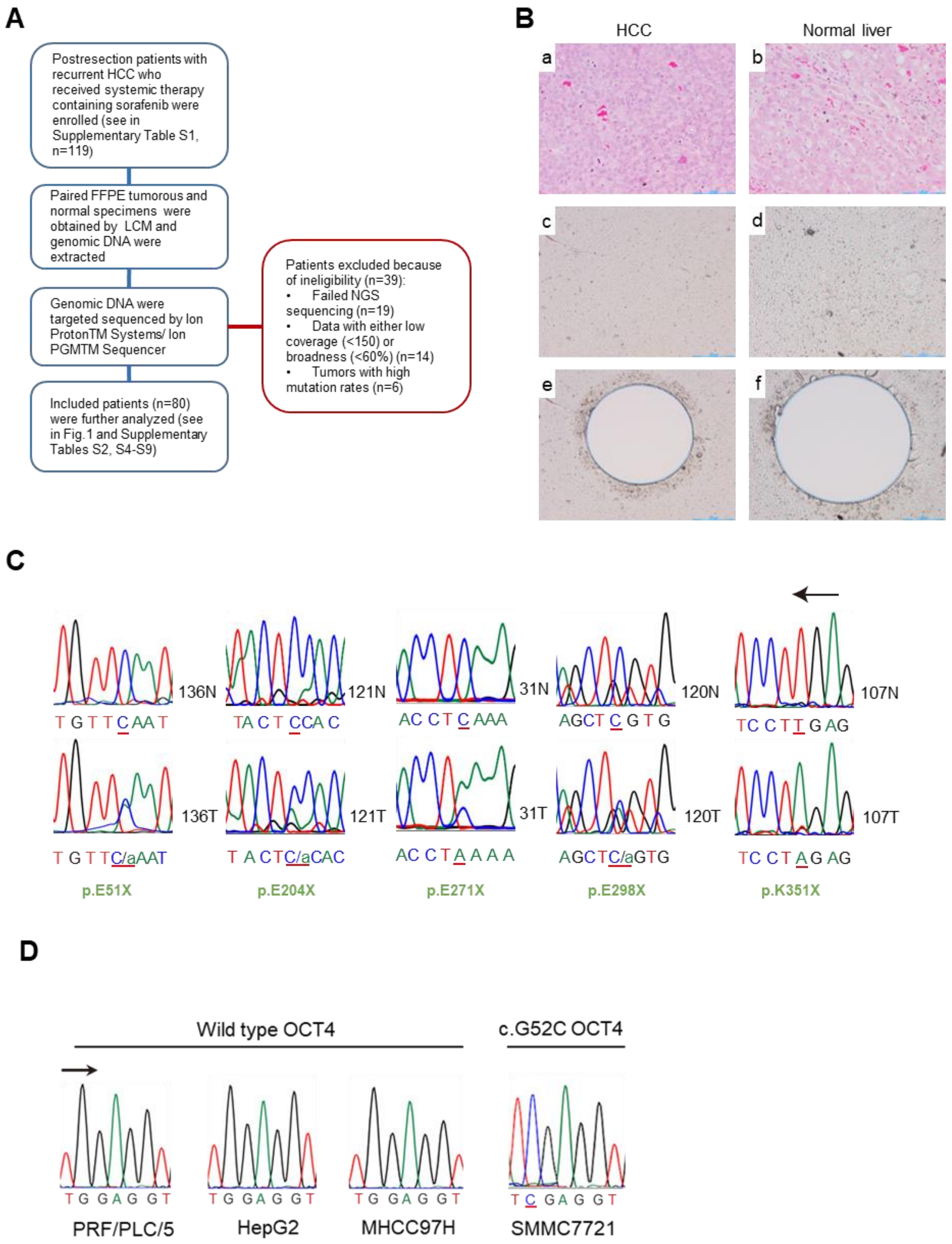

Supplementary Figure 2

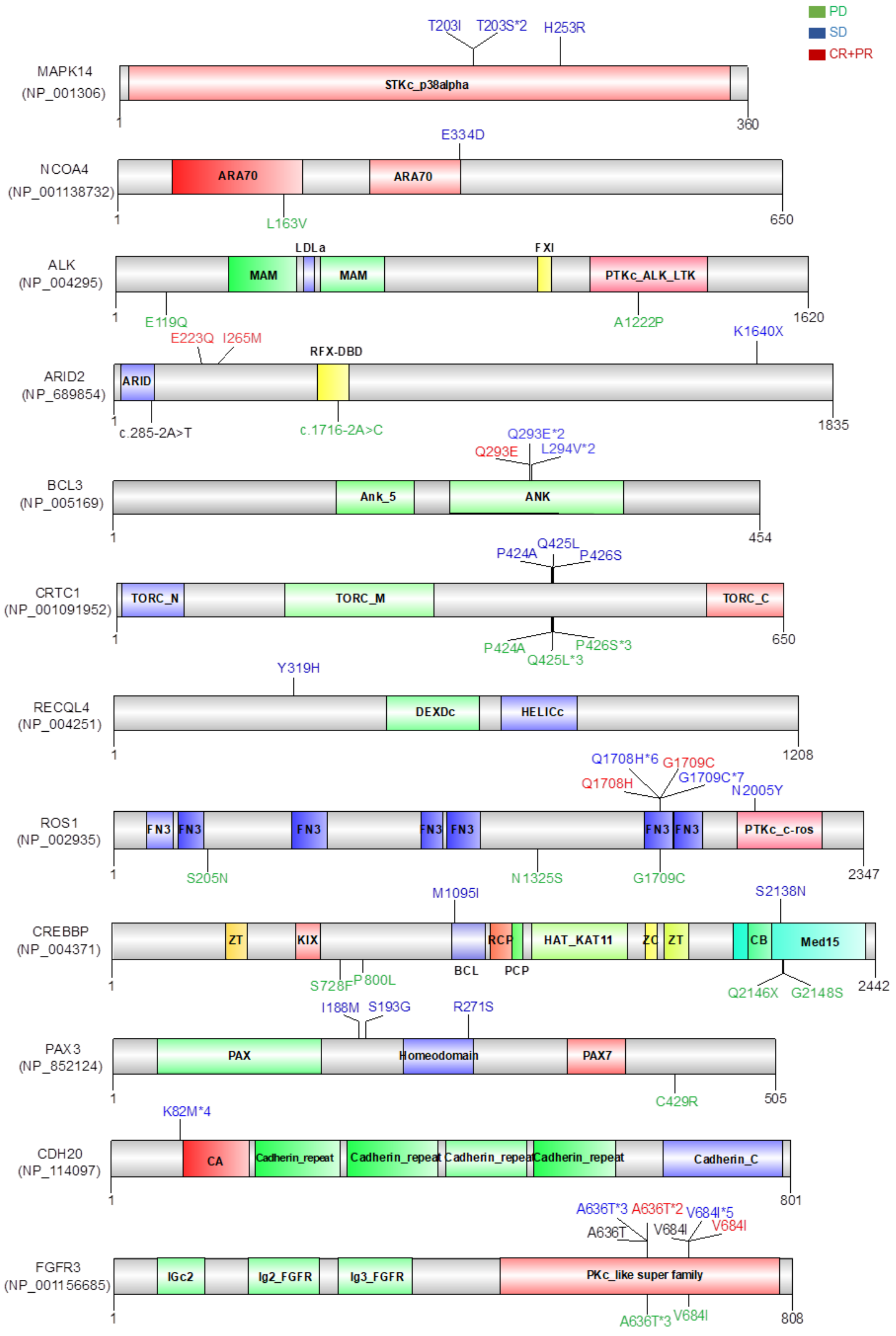

Supplementary Figure 3

A

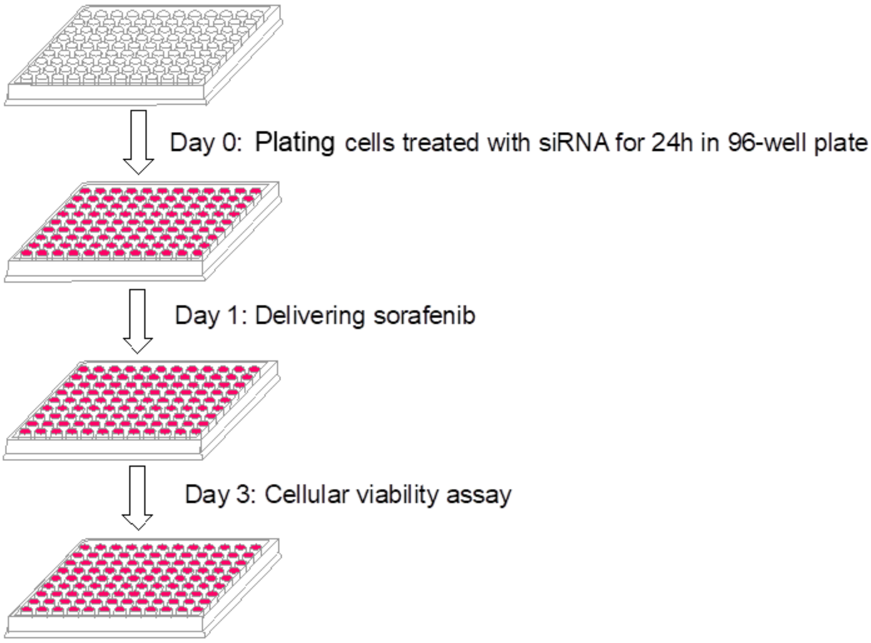

B

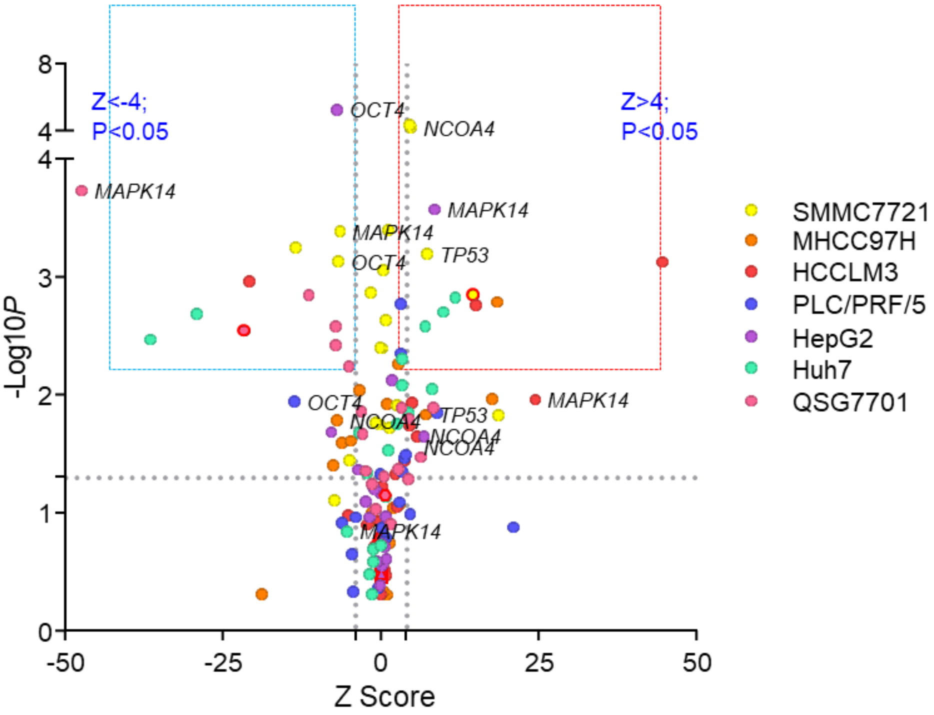

Supplementary Figure 4

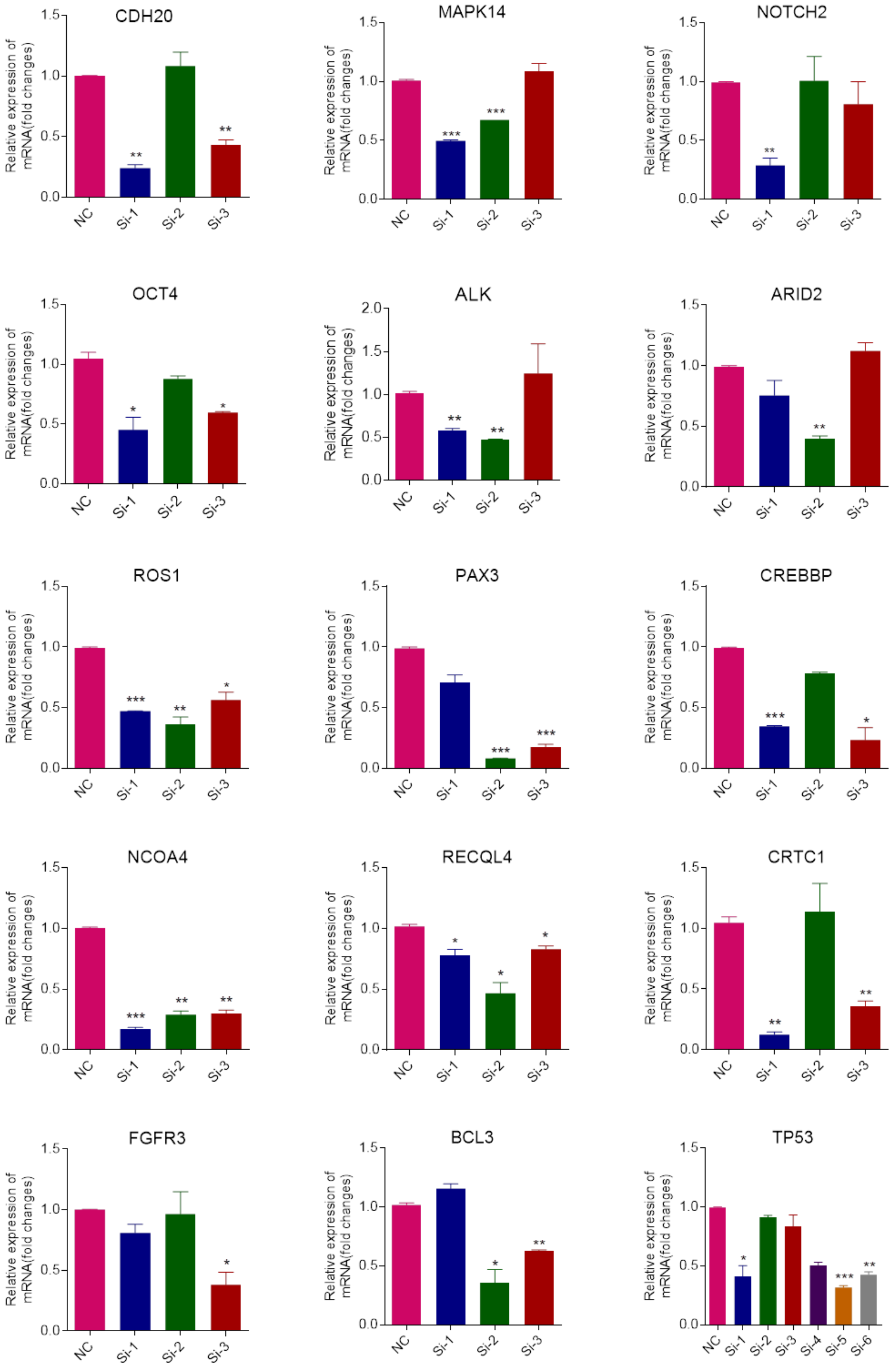

Supplementary Figure 5

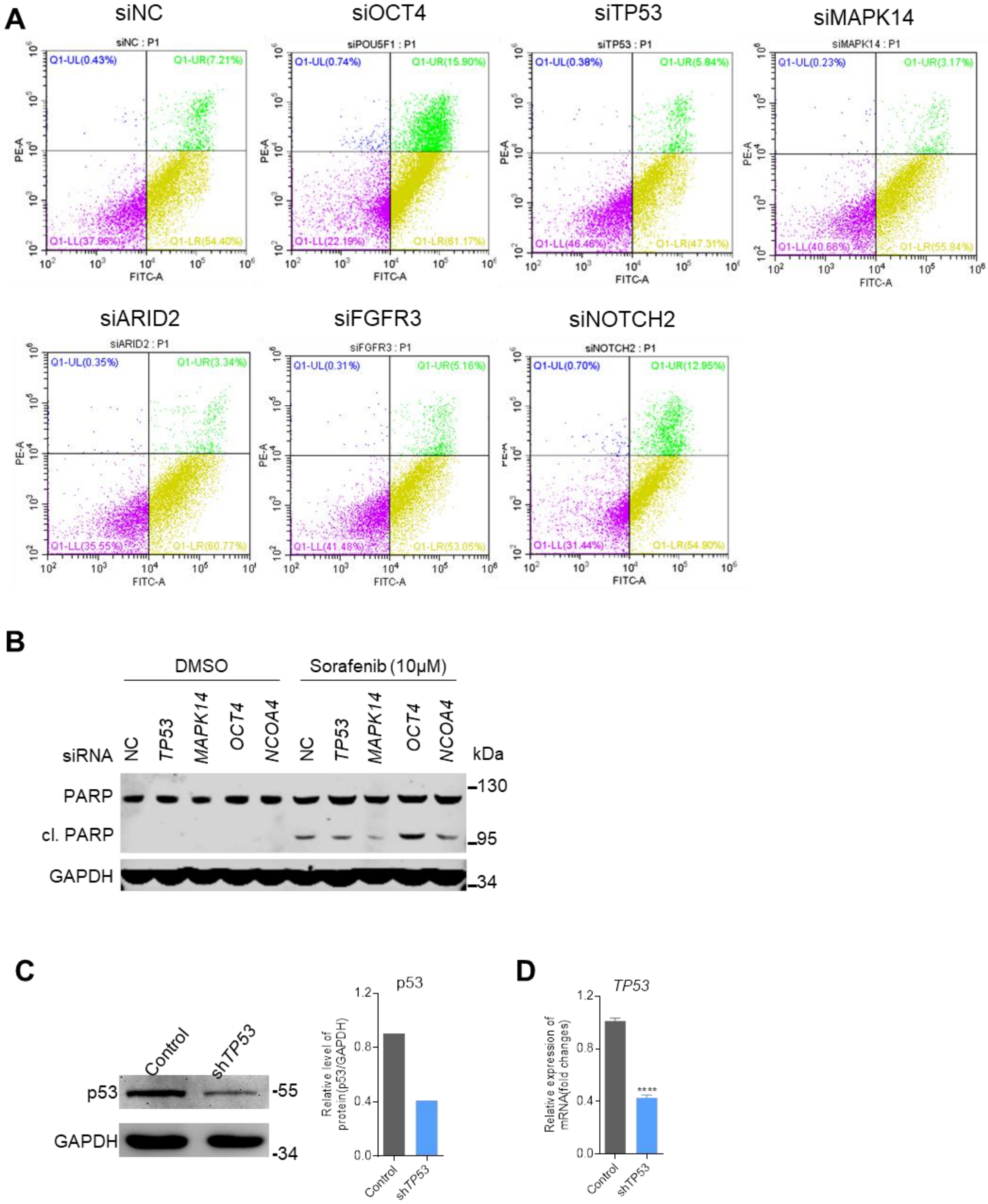

Supplementary Figure 6

A

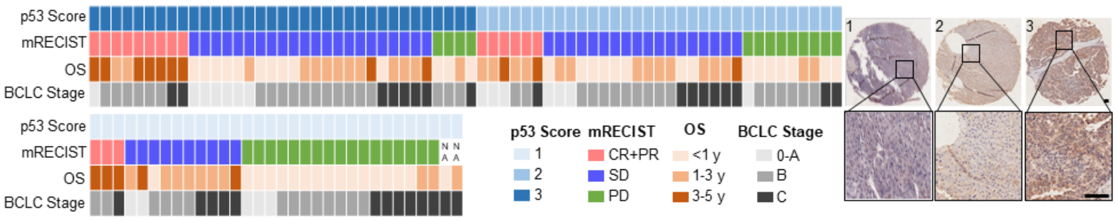

B

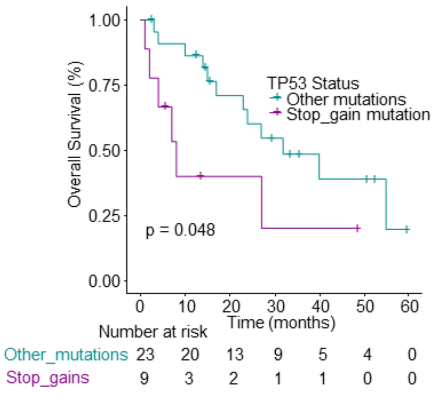

C

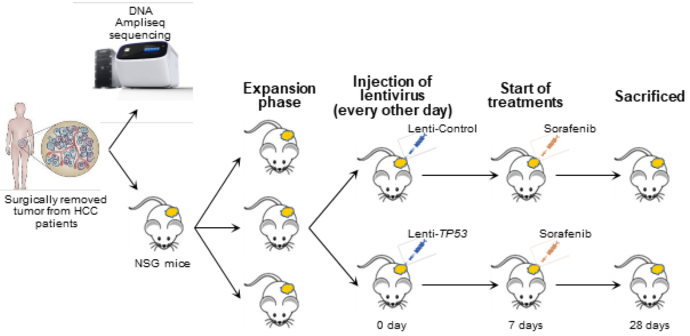

D

	p53			OCT4		
	**High**	**Low**	**P value**	**High**	**Low**	**P value**
Age≥60 years	2 (4.0)	2 (4.0)	1.000	1 (2.0)	3 (6.0)	0.307
Female	8 (16.0)	2 (4.0)	**0.046**	5 (10.0)	5 (10.0)	1.000
AFP>500 ng/ml	22 (44.0)	20 (40.0)	0.685	16 (32.0)	26 (52.0)	**0.043**
HBV infection	42 (84.0)	45 (90.0)	0.372	44 (88.0)	43 (86.0)	0.766
HBV DNA	17 (34.0)	24 (48.0)	0.155	25 (50.0)	16 (32.0)	0.067
HCV infection	2 (4.0)	1 (2.0)	0.558	1 (2.0)	2 (4.0)	0.558
Cirrhosis	18 (36.0)	23 (46.0)	0.309	16 (32.0)	25 (50.0)	0.067
Tumor size≥5 cm	35 (70.0)	31 (62.0)	0.398	39 (78.0)	27 (54.0)	**0.011**
Multinodular	12 (24.0)	8 (16.0)	0.317	9 (18.0)	11 (22.0)	0.617
Microvascular invasion	10 (20.0)	14 (28.0)	0.349	36 (72.0)	29 (58.0)	0.142
Macrovascular invasion	31 (62.0)	34 (68.0)	0.529	16 (32.0)	8 (16.0)	0.061
Tumor grade			0.695			0.240
I-II	3 (6.0)	4 (8.0)		2 (4.0)	5 (10.0)	
III-IV	47 (94.0)	46 (92.0)		48 (96.0)	45 (90.0)	

Data are shown as No. (%). Abbreviations: AFP, alpha fetoprotein; HBV, hepatitis B virus; HCV, hepatitis C virus.

Supplementary Figure 7

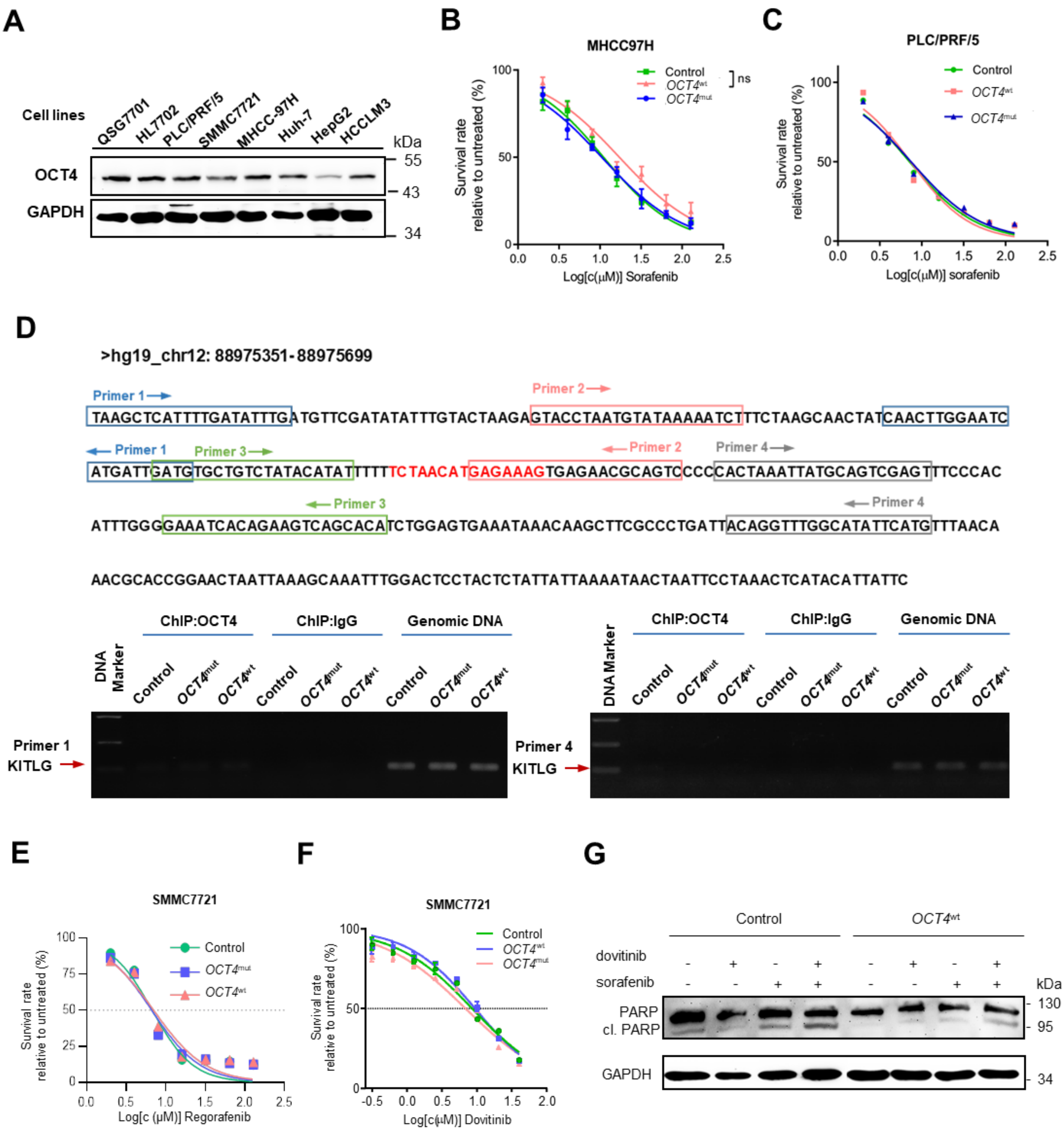

Supplement: Supplementary file 1 — Supplementary materials and methods, figures. [file thnov10p5384s1.pdf]
